# Automatic processing of multimodal tomography datasets

**DOI:** 10.1107/S1600577516017756

**Published:** 2017-01-01

**Authors:** Aaron D. Parsons, Stephen W. T. Price, Nicola Wadeson, Mark Basham, Andrew M. Beale, Alun W. Ashton, J. Frederick. W. Mosselmans, Paul. D. Quinn

**Affiliations:** a Diamond Light Source, Didcot, OX11 0DE, UK; b Research Complex at Harwell, Didcot, OX11 0FA, UK; c Department of Chemistry, University College London, 20 Gordon Street, London WC1H 0AJ, UK

**Keywords:** big data, multimodal, imaging, mapping, tomography

## Abstract

With the development of fourth-generation high-brightness synchrotrons on the horizon, the already large volume of data that will be collected on imaging and mapping beamlines is set to increase by orders of magnitude. As such, an easy and accessible way of dealing with such large datasets as quickly as possible is required in order to be able to address the core scientific problems during the experimental data collection. *Savu* is an accessible and flexible big data processing framework that is able to deal with both the variety and the volume of data of multimodal and multidimensional scientific datasets output such as those from chemical tomography experiments on the I18 microfocus scanning beamline at Diamond Light Source.

## Abstract 2

Multimodal chemical tomography is a technique that has to deal with a variety of big (>100 GB) datasets. Here, a novel method for approaching the analysis of such data using a Python-based big data solution is presented.

## 1. Big data processing: a solution for modern scientific data processing   

Modern scientific investigations are producing data at an increasing rate and volume, such that the number of processing hours required soon exceeds that which is feasible for a single user to spend processing them. This also detracts from the possibility of real-time analysis as the experiment progresses, so users often cannot assess how successful the experiment was until after they have left the beamline/facility. Ideally, the user should be able to view the processed data as the data collection progresses, enabling them to interactively guide the experiment.

The IBM big data and analytics hub classifies big data according to four criteria: volume, variety, velocity and veracity (IBM, 2014 ▸). For scientific applications such as multimodal chemical tomography, detailed in this article, we are primarily concerned with the volume and the variety of the data, but with extensibility to high velocity throughput in the near future. We also need the pipeline to be usable and extensible by as many scientists as possible.

Due to the volume of the data, we require such a pipeline to be able to exploit data parallelism by running across a cluster. This constraint also means that due to limits on RAM for each node (or a typical PC) we would want to avoid loading all the data at once and instead rely on disc I/O. We would also wish to preserve the raw data to be archived to tape for future analysis/validation checks.

Unfortunately, such criteria exclude a lot of the most recently published candidates from use (Daurer *et al.*, 2016 ▸; Prescher & Prakapenka, 2015 ▸; Vogelgesang *et al.*, 2016 ▸; Liu *et al.*, 2012 ▸).

One of the more likely recent candidates for such a tool is *MMX-I* (Bergamaschi *et al.*, 2016 ▸). However, whilst it can deal with a reasonable variety of data, *MMX-I* is written in Java and limited to a single compute node. This places a limit on both the volume and the velocity of the data that can be processed. Java is also not broadly written among scientists, making it hard for beamline staff to access. *aXis2000* (aXis2000, 1997 ▸) is also limited for similar reasons.

*TomoPy* (Gürsoy *et al.*, 2014 ▸) is another leading candidate for scientific data processing for high-volume data. It is, however, a very topic-specific framework focused on tomography. This limits its possible applications, as it often requires a sizeable effort to extend it to include other data reduction processes such as X-ray fluorescence tomography (Hong *et al.*, 2015 ▸; Gursoy *et al.*, 2015 ▸) or even other tomography toolboxes (Pelt *et al.*, 2016 ▸). As it is highly optimized for use on particular cluster architecture it is hard to roll out a performant framework to different facilities.

### 1.1. *Savu*: an open-source Python-based scientific data processing pipeline   

*Savu* (https://github.com/DiamondLightSource/Savu) is an open-source processing pipeline, capable of processing multimodal, *n*-dimensional data in serial or parallel, on a PC or across a cluster. Developed at Diamond Light Source, it is an object-oriented Python framework that performs a list of processing steps as specified by the user (a ‘processing chain’) and it is targeted at dealing with a broad variety of scientific problems (Wadeson & Basham, 2016 ▸).

The processing chain is built by the beamline staff/user prior to execution, currently using a command line tool: a GUI is in development. Plugins are added to the chain and the parameters set *via* this tool, which is then recorded as a configuration in a NeXus-formatted file. Once a configuration file is crafted, the user can run successive datasets through this processing chain automatically. This is particularly useful for experiments which yield a high number of datasets with similar, or the same, processing requirements, such as those detailed in the work presented here. During experiments in which this processing chain changes, users have been able to follow the documentation to easily amend the parameters.

Each step of the chain is implemented in a modular ‘plugin’ format that is abstracted from the rest of the framework, making plugins very easy to write, extend and use. The framework can be separated into three distinct layers: the plugin layer, the data layer and the control layer.

#### 1.1.1. The plugin layer   

The plugin layer performs the actual processing of the data. Each plugin performs a specific, independent task, such as filtering or tomographic reconstruction, which can be applied on the central processing unit (CPU) or graphics processing unit (GPU). The plugin task must be parallelizable across a data dimension, where the smallest potential unit of data parallelism is known as a frame. A frame can be any shape or rank and is referenced by setting a ‘pattern’ keyword that describes the data. Plugins need only specify which pattern they will input and output, and the framework organizes the rest.

The plugin layer is not concerned with the transport of the data, which allows plugins to be easily slotted into the existing framework. Each plugin simply requests the type and amount of data it requires, and the framework organizes the movement of these chunks of data (where a chunk is one or more frames). It is the job of the plugin to process the requested frames and return the output to the framework. The key result of this abstraction is that the scientist need only contribute the part of the code that performs the process, which usually they already have in the form of a script.

There are also two special categories of plugins known as loaders and savers, which must start and end the processing chain, respectively, allowing a variety of different data formats.

#### 1.1.2. The data layer   

The data layer holds all the information relating to each of the datasets associated with the current processing chain. The framework is capable of holding (and processing) multiple datasets at a time, creating and deleting them as the processing chain is traversed. Each dataset can experience its own unique list of processing steps because only the desired plugins need to be applied to each dataset and *vice* *versa*. The data layer also deals with the data slicing (*i.e.* organizes and extracts the relevant data in the order required by the plugin), data padding as well as organization of the associated metadata, *i.e.* axis information.

#### 1.1.3. The control layer   

The control layer runs and controls the processing chain, as well as the interaction between the plugin layer and the data layer. It is responsible for the management of available datasets and controls the passing of data to the plugin.

#### 1.1.4. Backends   

The data and the control layers encompass the movement and access of the data with further abstraction, creating a sub-layer known as the transport layer. The transport layer interfaces the plugins to the different ‘backends’: a term used here to describe the mechanism responsible for data parallelism. The framework is designed to allow easy extraction of the transport layer, allowing interchangeable backends, and currently offers Parallel HDF5 (The HDF group, 2014*a* ▸) and DistArray (Enthought Canopy, 2015 ▸), providing a trade-off between memory and speed. Parallel HDF5 is particularly useful for large datasets, as the data can be accessed by parallel reads/writes directly to/from a file, but speed is limited by MPI (Message Passing Interface) I/O. DistArray distributes the data across the processes, providing fast access to the data during processing, but memory is limited to available RAM.

Current backends under investigation include HDF5 virtual dataset (The HDF group, 2016 ▸) and HDF5 SWMR (The HDF group, 2014*b* ▸).

It is useful to note here that the package dependencies of *Savu* are merely MPI, the Anaconda distribution of Python (Anaconda, 2016 ▸) containing the packages relevant to the desired plugins and the dependencies necessary for the backend.

## 2. Application: multimodal chemical tomography   

Biological and material science problems are often investigated by a single microscopy/spectroscopy technique. However, such investigations using a single technique do not provide the complete solution to the problem. Therefore, it is often beneficial and instructive to collect data from multiple modalities simultaneously (Price *et al.*, 2015*a* ▸,*b* ▸). The continuing development of chemical tomography techniques, yielding spatially resolved information on, for example, elemental and phase distribution, is able to provide a more detailed picture of the nature of a material than the corresponding bulk measurements.

### 2.1. Catalyst investigation   

Catalytic activity and selectivity are determined not just by the chemical composition of the catalyst but, in the case of supported catalysts, the distribution of the catalyst on the support. Conventional bulk measurement techniques such as X-ray fluorescence (XRF), X-ray diffraction (XRD) and X-ray absorption spectroscopy are able to determine the catalyst (chemical) composition; however, the information provided is an average of all components detected. The combination of these X-ray characterization techniques with computed tomography (CT) enables the location of chemical components within or on a support. This spatially resolved data provides superior information on the nature and location of the active state of the catalyst, the nature and stability of the support, and any changes that may occur during pre-treatment and activation.

#### 2.1.1. An example catalyst sample   

This specific example is a metal nanoparticle catalyst supported on graphitic carbon. Graphitic carbon is the support of choice for many precious metal catalysts. Importantly the precious metal can be readily reclaimed by combustion of the support and the carbon is inert under acidic and alkaline conditions. Furthermore, the precious metal precursors can be reduced at low temperatures (often at room temperature) in flowing hydrogen. However, graphitic carbon is less frequently employed as a support for base metals because these normally require higher calcination temperature for graphitization, often leading to oxidation of the support material, or else in the presence of hydrogen at relatively high temperatures leads to hydrogenation of the support material; oxidation and hydrogenation often resulting in the collapse of the carbonaceous support. Graphitic carbon is produced by pyrolysis and subsequent graphitization (*e.g.* with steam) of naturally occurring carbon sources (wood, peat, nutshells), after which a catalytic precursor in the form of a soluble metal salt (often a metal nitrate) is applied *via* impregnation. Calcination and reduction procedures are subsequently employed to produce the final catalyst. The catalyst studied here was synthesized by a novel method to obtain carbon-supported base metal nanoparticles in a single pyrolysis treatment (Hoekstra *et al.*, 2015*a* ▸,*b* ▸).

#### 2.1.2. Techniques   

µXRF-CT reveals the location of elements in the particle but not their chemical structure or extent of interaction; that is, whether the elements are just co-located on the micrometre scale or in fact alloyed, what the particle sizes are and whether they are non-crystalline or crystalline. Besides the inability to resolve the chemical structure present, µXRF-CT measurements are limited by the energy of incident and fluorescent X-rays, *i.e.* if the incident X-ray energy is below the binding energy of a core electron of a given element, then the element will not be detected as no fluorescence will occur. Hence, the µXRF-CT signal is affected by the attenuation of the probe radiation. Conversely, if the energy of the fluorescent X-ray of an element is very low (or the sample density and volume are sufficiently large) then the fluorescent X-ray will be attenuated and not able to penetrate the sample beyond a few micrometres and, therefore, not be representatively detected.

µXRD-CT on the other hand is not limited by the identity of the elements in the sample, rather the crystallinity and, therefore, provides a good complement to µXRF-CT, giving information on interaction of elements present (Beale *et al.*, 2014 ▸). Whilst crystalline structure is a necessity, the elemental mass is less important; thus µXRD-CT can detect much lighter/heavier elements than would be visible by µXRF-CT with a given incident X-ray energy. Absorption-CT enables the identification of pore structure and voids that may be present within a sample. These can appear as empty regions in both µXRF-CT and µXRD-CT.

However, when using these methods it may be that there is an element that is too light/heavy and/or in a physical state that does not possess ordering on the scale of the beam size which will be more difficult to detect. It is from the complementary information provided by an absorption measurement that this detail is revealed.

A secondary use of collecting absorption-CT with a corresponding µXRF-CT dataset is that the absorption data may be used to correct for sample absorption effects. This is often manifest as shadowing of the fluorescence signal on the side of the sample furthest from the detector, particularly if the element of interest has a low atomic mass, or the sample volume/density is large. However, even without obvious shadowing, correction for sample absorption improves the quality of the reconstructed image by improving quantification and distribution estimates. This same correction can also be used to correct for beam hardening of µXRD-CT data, but we do not pursue this in the current work.

The collection of these datasets in parallel not only reduces the duration (and hence required dose) of the experiment, but also guarantees that the sample is in the same state for each measurement, something of crucial importance when imaging a dynamic system, such as an industrial catalyst during operation.

For this particular sample, only a two-dimensional slice is shown; however, in many cases the structural information of interest will be three-dimensional. This, therefore, necessitates imaging of larger three-dimensional volumes at each stage rather than a time series of two-dimensional slices. As such, the processing pipeline must be able to handle large volumes of multidimensional data.

#### 2.1.3. Experimental   

The experiment was performed at the I18 microfocus spectroscopy beamline at Diamond Light Source (Mosselmans *et al.*, 2009 ▸).

A catalyst sample was loaded into a 400 µm outer diameter quartz capillary (10 µm wall thickness). The capillary was mounted on top of a motorized Gothic arch bearing stage with *XY* travel to allow for centring of the particle on the axis of rotation. µXRF-CT data was collected by a Vortex ME-4 silicon drifts detector and XSPRESS-3 electronics. The X-ray beam was focused to a 2.5 × 3.0 µm spot (V × H) using Kirkpatrick–Baez mirrors and the sample was rastered across the beam in a translate–rotate data collection scheme with a µXRF spectrum collected at 2 µm intervals with a collection time of 1.0 s per pixel. µXRD-CT data was collected concurrently with the µXRF-CT data with a collection time of 650 ms and 350 ms readout time per pixel. The images were recorded using a Photonic Sciences CMOS-based X-ray imaging detector. The detector was calibrated using a LaB_6_ reference material. Absorption-CT data was also collected at the same time by use of an ion chamber positioned behind the sample. Each sinogram consisted of *ca* 100 points per row. A total of 52 projections were imaged, in 7° rotational steps, which, although coarser than for a typical full-field tomography dataset, was sufficient to provide a good quality tomography reconstruction.

### 2.2. The processing chain   

**Figure 1:**
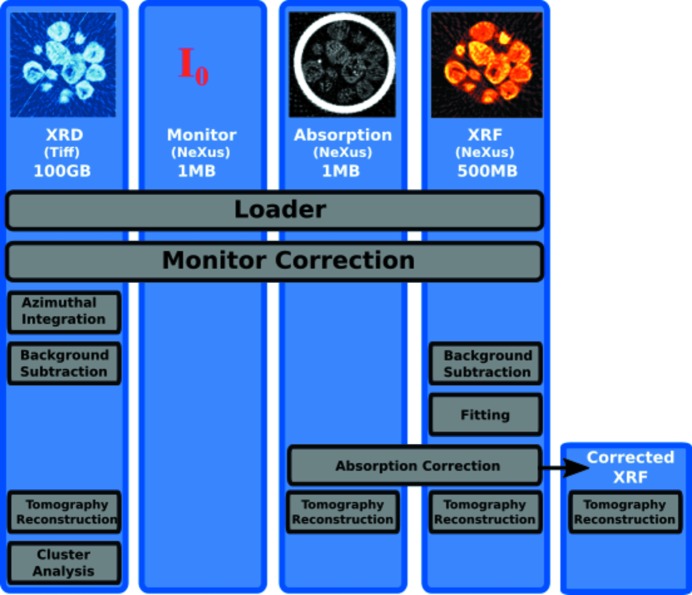
The processing chain used for this analysis. Four datasets are loaded from both NeXus-formatted HDF and a folder containing TIFFs. The images at the top are a reference for the output of the tomography stage of the processing described in this section. A new dataset is created when we perform the XRF absorption correction so that we may assess its impact on the output.

To process these different modes of data we must pass through a number of different reduction and filtering steps (Fig. 1 ▸). The mapping of the number of input to output data-sets can vary between one-to-one, many-to-one, one-to-many, many-to-many and one-to-none where metadata such as frame statistic can be added but no output data provided. As we will see in the following example processing chain, the plugin structure of *Savu* can deal with all of these mappings with no extra work required by users, and very minimal work required for plugin developers.

The two key parts to any processing chain are the methods for loading and saving data. In *Savu* these are represented as specific types of plugin of which there are a pre-written selection, or can easily be manufactured according to the data source.

#### 2.2.1. Loader   

For the processing chain used in this work, the data is mostly read in from NeXus (Könnecke *et al.*, 2015 ▸) formatted HDF5, whereby the different modalities are organized by application definitions. Whilst HDF5 format is preferential due to the sliceable and chunkable nature of their datasets, in this instance the XRD data is output from the detector in TIFF (tagged image file format) for which we make use of the functionality of FabIO (FabIO, 2016 ▸) to read. Hence, *Savu* currently supports all file types which are supported by FabIO, and is extensible to most other file types.

The output data and user-definable metadata from the processing is also output into HDF5 files *via* a standardized ‘saver’ plugin.

#### 2.2.2. Monitor correction   

The first step in the processing chain involves correcting the datasets by an *I*_0_ measurement. In the process list used for this work we do this in one of two ways. The first involves using a bespoke plugin, which takes the *I*_0_ dataset and another dataset and outputs a single, corrected dataset normalized to the input beam flux. Another way to do this would be to use a plugin that performs basic operations. This plugin can take in, and output, any number of datasets, and subjects them to basic linear operations (available from NumPy) by parsing an input string of the command. Here the former of these choices are used because it standardizes the procedure.

#### 2.2.3. Data reduction: XRD azimuthal integration   

**Figure 2:**
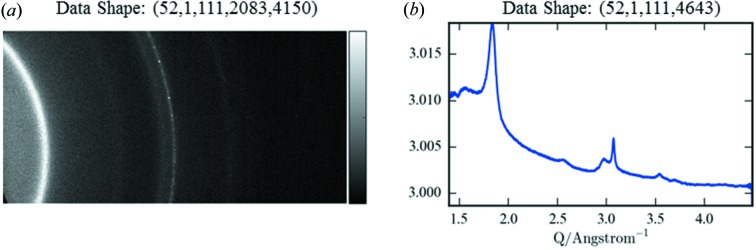
(*a*) Raw 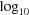 plot of two-dimensional X-ray diffraction image, (*b*) the corresponding calibrated and azimuthally integrated 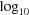 one-dimensional diffraction pattern.

The process to be applied to the data in the chain is to reduce the XRD images *via* azimuthal integration to representative patterns (Fig. 2 ▸). This is an example of a one-to-one I/O mapping, but where the data changes shape. Here, we have written a plugin using the CPU implementation of the ESRF package *pyFAI* (Kieffer & Karkoulis, 2013 ▸). This choice demonstrates one of the central tenets of *Savu*; that we should not reinvent the wheel for processes that already have heavily optimized solutions. The CPU version of *pyFAI* reduces each image into line profiles taking 300 ms per 2083 × 4150 frame. We rely on input from the user in the form of a NeXus calibration file for the detector geometry. In this instance, this is generated using *DAWN* (Basham *et al.*, 2015 ▸) *via* a LaB_6_ calibrant.

#### 2.2.4. Background subtraction   

The next step in the processing chain is to remove the background from all the data again in a many-to-many mapping because we take in both XRD patterns and XRF spectra and return the patterns/spectra minus the background. A plugin was written to do this which uses the same concept as that of the strip background method in *PyMca* (Solé *et al.*, 2007 ▸), whereby a moving wireframe window is averaged as it is iterated over the spectrum. One hundred iterations were used for each pattern/spectrum to remove the peaks. The resultant continuum was subtracted from the patterns/spectra before they were returned. The process takes around 20 ms per pattern/spectrum. This demonstrates the accessibility of *Savu*: it is very easy and quick to assemble prototype methods for testing. Other background subtraction methods are currently being implemented, for example, by directly including *PyMca* in a plugin.

#### 2.2.5. XRF curve fitting   

**Figure 3:**
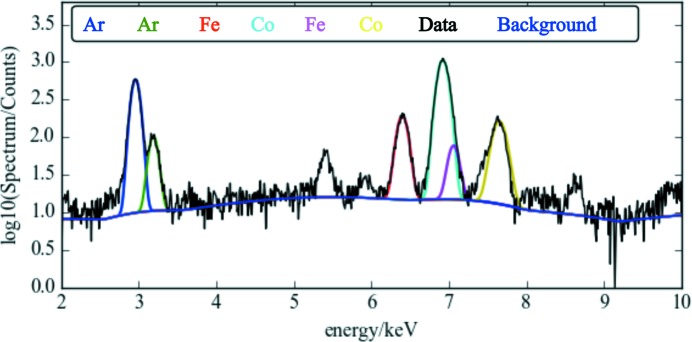
An example fit of the XRF pattern showing the underlying XRF peaks and background superposed onto the data.

The XRF spectra were fitted with Gaussian line shapes. The positions of the fluorescence lines, escape and sum peaks were calculated using the *xraylib* library (Schoonjans *et al.*, 2011 ▸). The detected rate of fluorescent photons was high, meaning that the dataset was subjected to pileup effects. To combat this during the fitting the peak width was refined in combination with the weights in order to take into account the peak broadening. Pileup peaks were also fit to improve the stability of the model. Fig. 3 ▸ shows an example fit and background subtraction for a fluorescent spectrum.

#### 2.2.6. XRF sample absorption correction   

One cause of artefacts in µXRF-CT stems from absorption of both the probe radiation and the softer fluorescent X-ray photons by the sample itself. Here we just consider the attenuation of the fluorescent X-ray photons. The fluorescent X-ray photon measured at a point on the sample furthest from the detector has a longer exit path through the sample compared with a point measured nearest the detector. Whilst this can be mitigated somewhat by the data collection strategy, it is common that the sample absorption may cause shadowing for some projections, leading to a reduction in contrast or, worse still, tails forming around high-density features (Ruiz-Martínez *et al.*, 2013 ▸).

**Figure 4:**
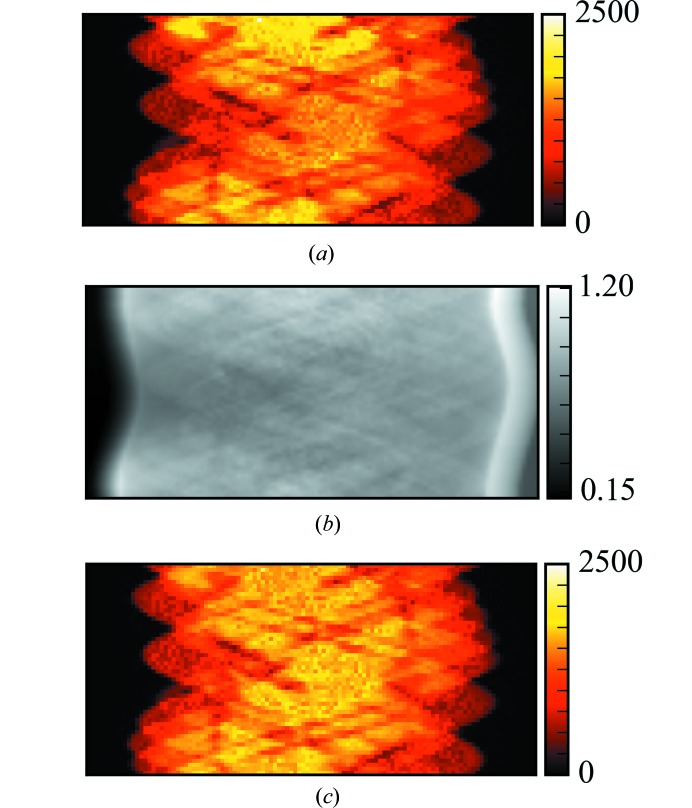
Sample absorption correction. (*a*) The sinogram of the cobalt *K*α peak area as collected. (*b*) The correction factor calculated from the absorption-CT dataset collected simultaneously. (*c*) The corrected cobalt *K*α peak area.

Whilst the high energy of the cobalt *K* lines, combined with the low metal loading and low-density support of the sample, very much reduce this effect for this sample, we demonstrate it is possible to correct for this in *Savu* by implementing a simple version of McNear’s absorption correction (McNear *et al.*, 2005 ▸). The absorption-CT data and the µXRF-CT data are both input into the plugin along with a user input of the sample composition, which is passed to *xraylib* to work out default parameters for the relative absorption coefficients at the XRF energies. The sample absorption corrections are calculated using the XRF fit areas and absorption in sinogram space, and are demonstrated in Fig. 4 ▸. A very slight change can be seen in the distribution of intensities. We generate a new dataset by branching the workflow at this point so that we can compare the impact of this correction at the end of the pipeline.

The implementation of this plugin demonstrates how, by abstracting away the data-handling processes, it is very easy for the scientist to move from techniques seen in a journal article to the process being implemented in an MPI-capable unit-tested framework.

#### 2.2.7. Tomography   

Each of the datasets is now passed through a tomography plugin to carry out the reconstruction. Currently in *Savu* there are a few different options available, including home-written methods for different algorithms using SciKit projections as well as the Astra Tomography Toolbox (GPU and CPU versions are available) (Palenstijn *et al.*, 2011 ▸; van Aarle *et al.*, 2015 ▸). Other versions may also be implemented and are straightforward to contribute *via* a simple extension.

For the work detailed in this paper we use a plugin implementing the GPU version of the Astra Tomography Toolbox filtered back projection (FBP) reconstruction routine to reconstruct the sinograms from all three data modalities.

**Figure 5:**
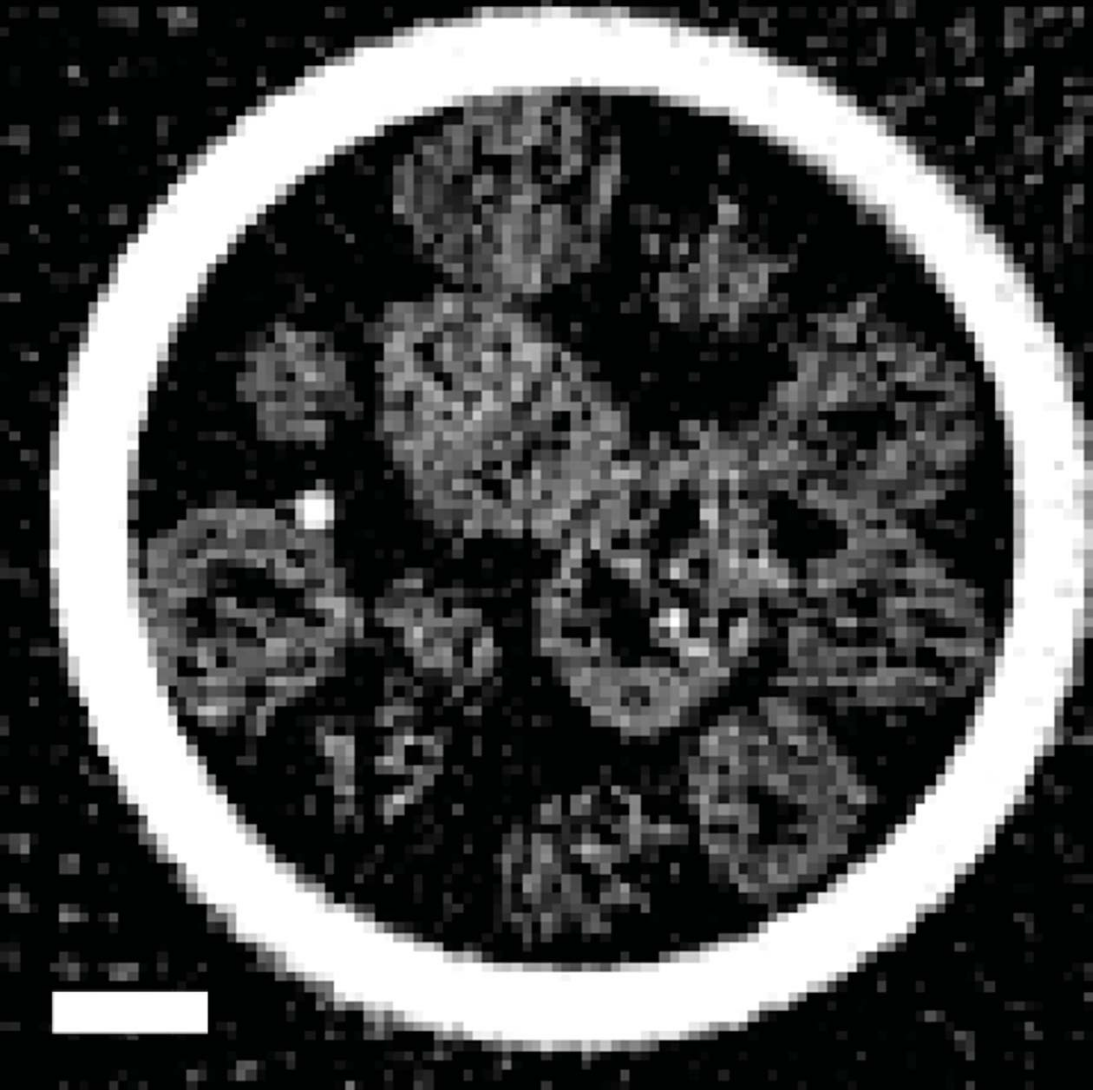
Tomography reconstruction using filter back projection of the absorption-CT data. The bright circle present in the reconstruction is the capillary wall. The scale bar represents 20 µm.

*Absorption-CT.* The absorption-CT was reconstructed as detailed above with the result shown in Fig. 5 ▸. Due to the reduced sampling of the dataset (a feature of the dwell times in the current experimental setup), some artefacts are seen in the corners of the reconstructed slice. These artefacts also cause issues with traditional auto-centring methods. Therefore, instead of automatically finding the centre we use the parameter scanning functionality in *Savu* to run the same reconstruction but with five centres of rotation. This adds an extra slice dimension to the data at the output, allowing us to quickly find the best value for the parameter, the best of which is shown in Fig. 5 ▸.

It should be added here that *any* user-exposed parameters can be scanned in this manner for all plugins. This includes being able to scan the reconstruction method across all those available in a suitable toolbox, *e.g.* the Astra Tomography Toolbox.

**Figure 6:**
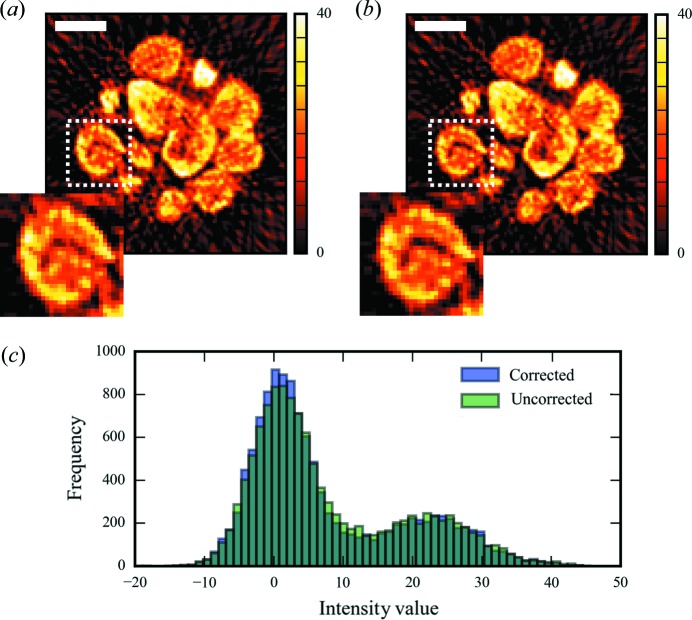
(*a*) Tomography reconstruction by filtered back projection of the Gaussian area of the fitted cobalt *K*α peak. (*b*) The reconstruction of the same data processed after absorption correction of the sinogram. Insets of (*a*) and (*b*) show a slight sharpening of the features in the absorption corrected image. The scale bar represents 20 µm. (*c*) Histograms of both corrected and uncorrected data showing a slight improvement in contrast.

*μXRF-CT.* The areas from both the absorption-corrected and the raw XRF fit are now also passed to the same plugin (without taking the log of the data), and the tomography reconstruction carried out as previously detailed. The results for the fit of the cobalt *K*α line are shown in Fig. 6 ▸.

As we branched the workflow at the point that the correction filter was applied, we can now assess its impact. At first glance it would appear that not much improvement is seen in the reconstruction as expected; there was not much shadowing in the un-corrected data due to the high energy of the XRF photons and the low density of the sample. However, on closer inspection we can see that there has indeed been a slight contrast improvement, and that pores features that represent voids in the support which are not visible in the uncorrected data are apparent in the corrected version. By looking at the histogram of the reconstructed data we can also demonstrate that contrast has very slightly improved. Here this is seen as a slight ‘sharpening’ of the peak around zero density.

*μXRD-CT*. At this stage, the XRD data has already been reduced to one-dimensional patterns and background subtracted, and if we wanted we could try to fit the data according to theoretical line-shapes. However, this is not necessarily the most efficient way to solve this problem initially. For the dataset under study here, the data is fairly complex, with many overlapping peaks, which leads to problems detecting peaks that appear as ‘shoulders’ to other peaks. This is in part due to the nature of a tomographic dataset in that the pattern which we measure is a projection and, hence, an integrated version of not only the many phases present but also the contribution along an entire ray through the sample.

As an alternative approach we look to simplify the datasets by first performing the tomography reconstruction for each sinogram in the spectrum stack (4150 bins, each processed in parallel), using the GPU version of the Astra Tomography Toolbox implementation of FBP. This is a reasonable approach because the data in this case exhibit little ‘spottiness’; that is, we are in the approximation where the beam is much larger than the average crystallite size, with the result that our two-dimensional diffraction data contain smooth powder rings.

**Figure 7:**
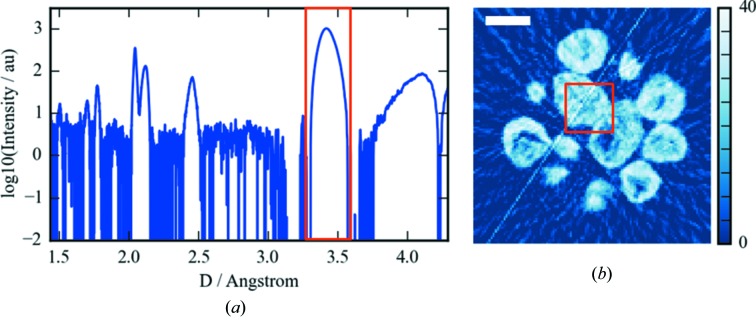
(*a*) Reconstructed µXRD-CT slice of the windowed area of the spatial spectrum shown in (*b*). (*b*) An averaged µXRD pattern over the area inside the red box in (*a*). The scale bar represents 20 µm.

The result from this is shown in the hyperspectral plot in Fig. 7 ▸. This part of the data reduction, although it has increased the size slightly, has simplified the signal per voxel.

We can now isolate the contribution by windowing or fitting individual peaks in the one-dimensional pattern domain and viewing them in the volume domain as shown in Fig. 7 ▸. However, because for this particular dataset we are not primarily concerned with the quantitative information about each phase, but more the distribution of the phases present with respect to each other, we prefer instead to apply a cluster analysis.

#### 2.2.8. Principal component analysis   

Principal component analysis (PCA) is a technique for clustering data that has begun to be increasingly more used in scientific data processing and has a dominant position in the business world (Jolliffe, 2002 ▸). The technique provides quantitative information about the distribution of data that can be organized into groups. In our case, this data is µXRD-CT patterns, and we use it to reduce the complexity to more clearly define areas of similar crystalline composition.

**Figure 8:**
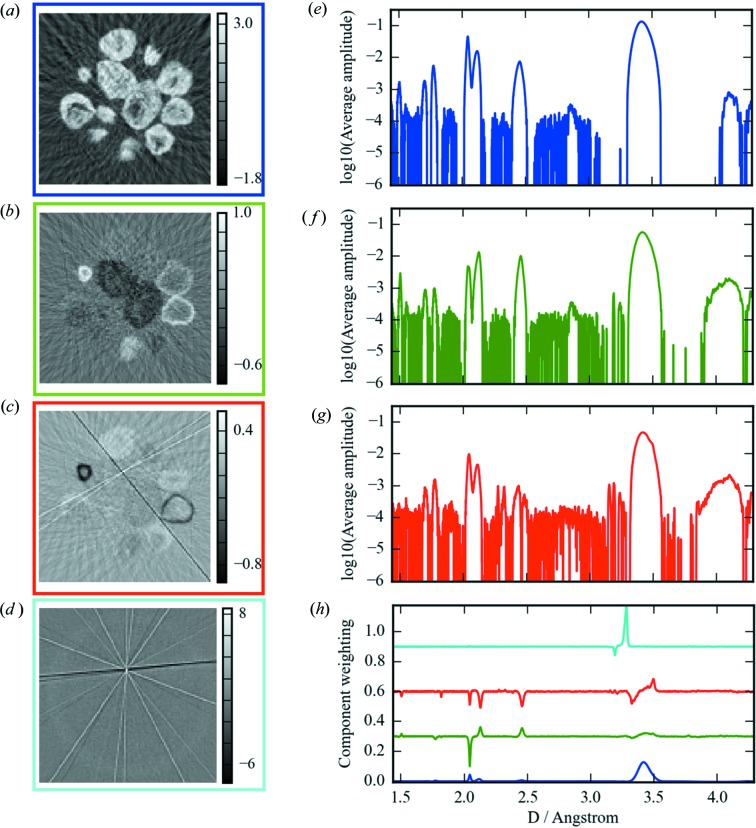
The results of the PCA analysis of the reduced µXRD patterns. (*a*)–(*c*) The loadings for the first three components. These show the three main clusters of the data. Panel (*a*) shows the mean average, panels (*b*) and (*c*) show the main deviations from it. Panel (*d*) shows the sum of the remaining loadings. These are indicative of a single-crystal artefact in the data, which the PCA has identified. The scores for these states are shown in (*h*). The loadings were then thresholded and applied as a mask to the tomography result in order to give representative µXRD patterns of each region identified. Panels (*e*)–(*g*) show these results next to their respective spatial clustering. Panel (*g*) shows a definite shoulder appearing on the large graphite peak at 3.42 Å. This suggests an expansion of the support. Panels (*f*) and (*g*) also show very different composition, the former being mostly in CoO, whereas the majority of the particles imaged are a mixture of CoO and metallic Co.

For our first implementation in *Savu* we directly use those methods that are available in the scikit-learn package for Python. This implementation took six lines of Python code, and as we can see from Fig. 8 ▸, has produced two instantly interesting and intuitive results.

Firstly, we can see from the loadings that the sample has two main detectable components [Figs. 8 ▸(*a*) and 8(*b*)]. By thresholding these loadings and applying them back as a mask on the XRD-CT dataset in Fig. 7 ▸, we can reduce the data down to a few clusters of similar crystalline composition, and sum the XRD patterns from each of these clusters, greatly reducing the amount of data and simplifying the interpretation. Three main crystallographic phases are identifiable as graphitic carbon (3.40 Å), CoO (1.51, 2.13, 2.46 Å) and cubic Co (1.77 and 2.05 Å). It is also possible [Figs. 8 ▸(*c*) and 8(*g*)] to see the expansion of the graphite support appearing as a shoulder peak to the main graphite peak at 3.42 Å.

The second useful result is that streak artefacts have been filtered out from the clusters (Fig. 8 ▸ *d*). These are caused by the presence of single crystals of similar scale to the incident beam. Nanocrystalline samples will produce diffraction rings at all rotations measured, whereas the single crystals will produce diffraction spots at certain rotations corresponding to the rocking curve of the crystal which, when azimuthally integrated and reconstructed, result in streaks across the reconstruction. Traditionally these peaks would be filtered out of the raw, unreduced XRD patterns, which is very time consuming. Here they come out of the data analysis as a by-product. It would be possible to use this information to index the single-crystal at this point; however, it was not of interest for the present experiment.

Although just a first approach at clustering, we have demonstrated that *Savu* can not only implement PCA but that it can be directly useful in the interpretation of the data.

## 3. Summary   

The described processing chain has been used to demonstrate the flexibility and power of the multimodal processing aspect of the *Savu* framework. This particular configured processing chain is currently in regular use at the I18 microfocus spectroscopy beamline at Diamond Light Source, with further required plugins being developed for use on the beamlines I14 and I13. The total run time for the processing exhibited here was around 10 min over two nodes of COM10 of the Diamond cluster. This cluster comprises of 12 Dell PowerEdge C4130 HPC nodes, each containing 2× Intel Xeon E5-2650 v3 @ 2.30 GHz which support 20 threads each. Each node has 256 GB RAM (DDR4 @ 2133 MHz) and 2× nvidia Tesla K80 GPGPUs. The file system is a GPFS (general parallel file system) networked to the cluster *via* FDR infiniband (Mellanox Technologies MT27500 Family [ConnectX-3]) (56 Gbits s^−1^). This cluster specification was chosen to give the best total FLOPS per GBP across the cluster rather than maximizing the performance of each element.

The time taken to collect this dataset was 90 min. The processing chain demonstrated here is under test using both Travis and Jenkins continuous integration, and is available here: https://github.com/DiamondLightSource/Savu/blob/master/test_data/process_lists/multimodal_tomo_i18.nxs. We are currently investigating the process to make it available *via* the Anaconda Python distribution as well as Docker. The total coverage of the Travis tests at the time of writing is 76% of the available code with an overall health of 84%.

One of the next stages in the development of *Savu* is to implement a HDF5 SWMR/Virtual dataset backend, which will allow the processing to happen as the data is being collected. This will pave the way towards truly real-time processing. We also plan to implement *PyMca* for the fluorescence fitting as well as provide integration for various packages for coherent X-ray imaging processes such as ptychography and coherent diffractive imaging. The pipeline will also be interfaced with the existing *DAWN* processing perspective, allowing users to switch between both with minimal effort.

## 4. Conclusions   

In conclusion, we have demonstrated that *Savu* is an easy to use plugin-based tool for multimodal big-data processing. By abstraction, the underlying data transport is hidden from the scientific developer/user which makes prototyping new processing steps easy and allows the integration of all Python-based software libraries with minimum effort. The software is open source and is readily applicable to all cluster architectures. Existing backends for live data processing are currently being implemented.
